# Genes associated with agronomic traits in non-heading Chinese cabbage identified by expression profiling

**DOI:** 10.1186/1471-2229-14-71

**Published:** 2014-03-22

**Authors:** Xiaoming Song, Ying Li, Tongkun Liu, Weike Duan, Zhinan Huang, Li Wang, Huawei Tan, Xilin Hou

**Affiliations:** 1 State Key Laboratory of Crop Genetics and Germplasm Enhancement/Key Laboratory of Biology and Germplasm Enhancement of Horticultural Crops in East China, Ministry of Agriculture, Nanjing Agricultural University, Nanjing 210095, China

**Keywords:** Non-heading Chinese cabbage, Expression profile, Differentially expressed genes, Protein function annotation, Chromosome distribution, Agronomic traits

## Abstract

**Background:**

The genomes of non-heading Chinese cabbage (*Brassica rapa* ssp. *chinensis*), heading Chinese cabbage (*Brassica rapa* ssp. *pekinensis*) and their close relative *Arabidopsis thaliana* have provided important resources for studying the evolution and genetic improvement of cruciferous plants. Natural growing conditions present these plants with a variety of physiological challenges for which they have a repertoire of genes that ensure adaptability and normal growth. We investigated the differential expressions of genes that control adaptability and development in plants growing in the natural environment to study underlying mechanisms of their expression.

**Results:**

Using digital gene expression tag profiling, we constructed an expression profile to identify genes related to important agronomic traits under natural growing conditions. Among three non-heading Chinese cabbage cultivars, we found thousands of genes that exhibited significant differences in expression levels at five developmental stages. Through comparative analysis and previous reports, we identified several candidate genes associated with late flowering, cold tolerance, self-incompatibility, and leaf color. Two genes related to cold tolerance were verified using quantitative real-time PCR.

**Conclusions:**

We identified a large number of genes associated with important agronomic traits of non-heading Chinese cabbage. This analysis will provide a wealth of resources for molecular-assisted breeding of cabbage. The raw data and detailed results of this analysis are available at the website http://nhccdata.njau.edu.cn.

## Background

*Brassica rapa* L. plants have a rich morphological and genetic diversity, comprising many plant subspecies that humans farm on an enormous scale worldwide. Examples of agriculture crops include turnip, field mustard, and Chinese cabbage. Non-heading Chinese cabbage (*Brassica rapa* ssp. *chinensis*), with its five varieties, is an excellent model to study the genetics and mechanisms underlying phenotypic diversity. Of particular interest are its flowering and self-incompatibility characteristics. In general, a higher plant’s conversion from vegetative growth to flowering is a pivotal point in ontogeny and decides the timing and quality of its reproduction. Floral induction has become a focus of research in *Brassica* vegetables [1-3], and non-heading Chinese cabbage is an important tool in this regard.

Recent progress in molecular biology techniques has revealed that floral induction is regulated through long-day, autonomous, vernalization, and gibberellin-dependent genetic pathways [4-6]. Early- and late-flowering mutants have been identified in the model plant *Arabidopsis*, and many key genes controlling flowering have also been isolated in other plants, genes that include *FLC*, *LFY*, *FT*, and *SOC1*[6-8]. However, there were few reports about the flowering of the non-heading Chinese cabbage, or the genes that regulate flowering.

Self-incompatibility is a genetic mechanism that prevents self-pollination (selfing) and inbreeding with close relatives. It promotes the divergence of species; any allele that is rare in the population has an advantage if residing in a plant that cannot self-fertilize. In angiosperms self-fertilization is prevented by the chemical recognition of pollen by pistils, which depends on self-sterile (*S*)-alleles in pollen or stigma, and has evolved independently at least three times [9]. However, the shift from outcrossing to selfing is a common evolutionary trend in higher plants related to the loss of function under natural selection of the *S*-alleles in pollen or stigma [10]. Thus, the plant self-incompatibility system is an excellent model for understanding the variability in S loci. Shifts between outcrossing and selfing and frequency-dependent selection leads to the long-term maintenance of many alleles with different incompatibility types [11], and alleles thus become widely dispersed throughout different populations of species [9,12].

In non-heading Chinese cabbage production, the self-incompatibility system is relied on for breeding, and also makes the plant a model system for studying reproductive biology and balancing selection [13]. With the recent development of next-generation, high-throughput sequencing technologies, the expression profiles of many species have been extensively studied. In addition, digital gene expression tag profiling has been used to study changes in gene expressions [14,15], giving a comprehensive snapshot of changes in mRNA expression that occur during biological processes. Expression levels can be calculated by the number of detected tags, and this information can facilitate our understanding of plant genetics and developmental mechanisms.

As of yet there has been no report of an expression profile for non-heading Chinese cabbage. To identify differentially expressed genes (DEGs) among different non-heading Chinese cabbage accessions in their natural environment, we conducted an expression profile analysis for plants growing under non-controlled conditions. Among the five varieties of non-heading Chinese cabbage produced in China, approximately 80% is Pak-choi (also known as bok-choy). Therefore, we chose three accessions of Pak-choi (NHCC001, NHCC002, and NHCC004) for investigating the differences in expression profiles, at five important developmental stages. Relative to the accessions NHCC001 and NHCC002, NHCC004 bolts and flowers later, and NHCC002 is the only one that is self-incompatible. The leaf color of NHCC002 is lighter than that of NHCC001 and NHCC004. We systematically and comprehensively evaluated the expression profiles of these accessions, to identify the DEGs at the five development stages, to analyze their expression patterns, and to identify candidate genes associated with important agronomic traits.

## Results and discussion

### Expression profiling of non-heading Chinese cabbage

We used high-throughput sequencing to survey the gene expression patterns of three non-heading Chinese cabbage cultivars (NHCC001, NHCC002, and NHCC004) at five development stages (five leaf, rosette, adult, bolting, and flowering). A total of 55.45 million reads of raw tags were sequenced. After filtering, we obtained approximately 17.47, 17.97, and 17.77 million reads of clean tags for NHCC001, NHCC002, and NHCC004, respectively, of all five developmental stages combined (Additional file 1: Table S1). In these clean tags, 73.61% (12.85 million), 69.73% (12.53 million), and 68.18% (12.12 million) reads from NHCC001, NHCC002, and NHCC004, respectively, could be mapped to non-heading Chinese cabbage genes modeled from the NHCC001 draft genome, and 63.00% (11.00 million), 60.37% (10.84 million), and 57.98% (10.30 million) reads from the respective accessions could be mapped to unique genes (Table 1).

**Table 1:** Alignment of the expression profile read to the genome of non-heading Chinese cabbage

**Sample accession**	**Developmental stage**	**Total reads**	**Mapped reads**	**Uninq mapped reads**	**Mapped ratio (%)**	**Uniq mapped ratio (%)**	**Average mapped ratio (%)**	**Average uniq mapped ratio (%)**
NHCC001	Seedling	3 416 587	2 698 481	2 348 539	78.98	68.74	73.61	63.00
	Rosette	3 524 418	2 530 708	2 226 602	71.80	63.18		
	Adult	3 606 773	2 607 373	2 118 040	72.29	58.72		
	Bolting	3 426 539	2 642 635	2 254 999	77.12	65.81		
	Flowering	3 500 555	2 375 503	2 049 847	67.86	58.56		
NHCC002	Seedling	3 421 189	2 542 362	2 258 193	74.31	66.01	69.73	60.37
	Rosette	3 622 963	2 571 338	2 280 414	70.97	62.94		
	Adult	3 648 700	2 604 740	2 164 281	71.39	59.32		
	Bolting	3 692 773	2 713 241	2 320 313	73.47	62.83		
	Flowering	3 587 470	2 099 277	1 820 960	58.52	50.76		
NHCC004	Seedling	3 488 808	1 985 099	1 765 494	56.90	50.60	68.18	57.98
	Rosette	3 396 774	2 434 589	2 115 424	71.67	62.28		
	Adult	3 706 078	2 642 722	2 136 807	71.31	57.66		
	Bolting	3 549 009	2 574 520	2 189 878	72.54	61.70		
	Flowering	3 628 344	2 484 967	2 092 015	68.49	57.66		

A total of 29 101 genes were detected at the five developmental stages of the three non-heading Chinese cabbage accessions (Additional file 2: Figure S1). There were 15 251, 13 316, and 5869 expressed genes shared by all five development stages in NHCC001, NHCC002, and NHCC004, respectively. We also conducted a study of gene expression in the different accessions at each stage. A total of 13 546, 15 281, 15 029, 16 333, and 12 704 co-expressed genes in all three accessions were detected in the leaf, rosette, adult, bolting, and flowering stages, respectively (Additional file 2: Figure S2).

### Identification of DEGs in non-heading Chinese cabbage

**Figure 1:**
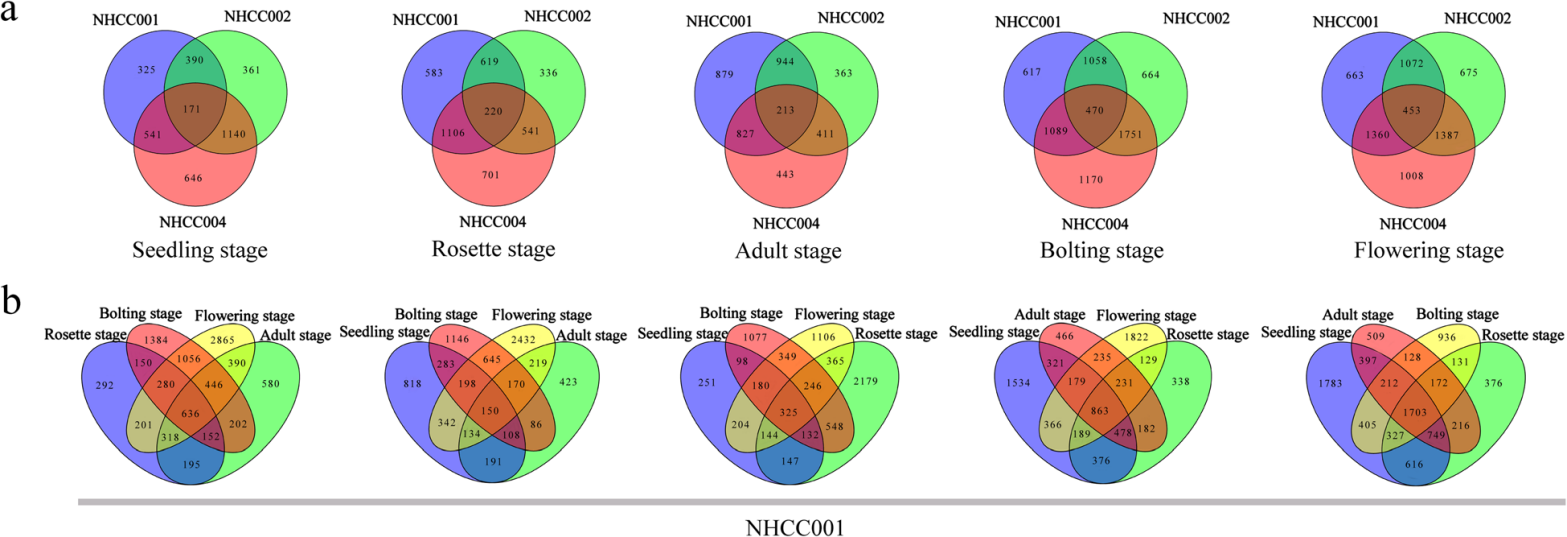
**The analysis of the differentially expressed genes. a)** Differentially expressed gene numbers for the three accessions at each developmental stage. **b)** Differentially expressed gene numbers for the five developmental stages in the NHCC001 accession.

A total of 15 830 unique genes were found to be differentially expressed among the three accessions in the five development stages (Additional file 1: Table S2). The number of DEGs per accession or developmental stage is shown as a Venn diagram and in tables (Figure 1, Additional file 2: Figure S3, Additional file 1: Table S3, Table S4). To gain insights into the DEGs, we conducted a chi-squared test, and the *P* values were corrected using the false discovery rate (Additional file 1: Table S5). The upregulated and downregulated genes are shown in a scatterplot (Additional file 2: Figure S4).

**Figure 2:**
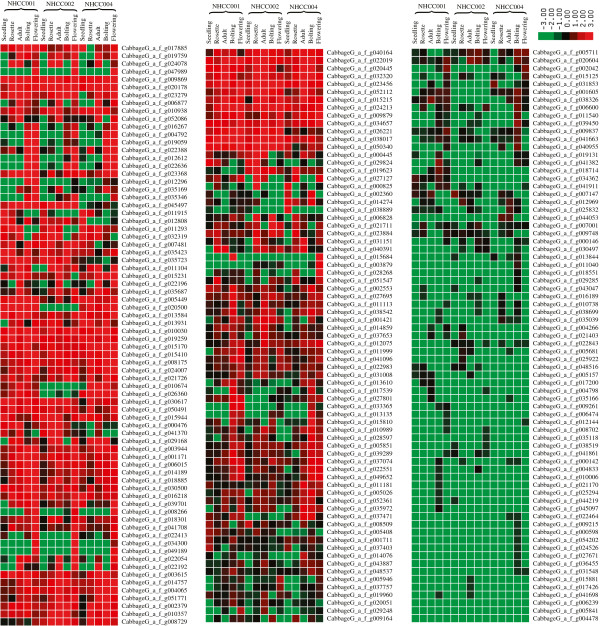
Cluster graph of flowering-time candidate genes, in TPM.

We used the Cluster program (http://bonsai.hgc.jp/~mdehoon/software/cluster/software.htm) to identify subgroups in the gene expression profiles that shared common features and had similar expression levels. We hypothesized that DEGs gathered in one group might have similar functions, or be involved in the same metabolic processes. In our analysis, clusters were plotted according to the DEG expression values. In Cluster, the DEGs that had similar expression levels were clustered together. By using these clusters, we could infer the function of newly identified genes according to the known genes in the same cluster, such as the cluster of flowering and self-incompatibility candidate genes (Figure 2, Additional file 2: Figure S5).

### Functional annotation and pathway analysis of DEGs

The cellular component, molecular function, and biological process associated with each of the DEGs were obtained using the Gene Ontology (GO) database. For example, there were more genes related to antioxidant activity, translation regulator activity, and reproduction in NHCC001 than in NHCC002 at the seedling stage. However, there were more auxiliary transport genes in NHCC002 than in NHCC001 (Additional file 2: Figure S6). The genes that belonged to the same orthologous cluster were classified into one group, based on the Clusters of Orthologous Groups (COG) database. Taking the DEGs of NHCC001 and NHCC002 at the seedling stage as an example, the cluster results showed that most DEG genes belonged to the general function category, followed by genes related to translation, ribosomal structure, and biogenesis (Additional file 2: Figure S7). Kyoto Encyclopedia of Genes and Genomes (KEGG) pathway analysis was performed to elucidate the energy metabolism, signal transduction, and biological systems of DEGs. We found that several DEGs are involved in several important pathways for plant growth and development, such as flowering genes and chlorophyll gene pathways, described in detail below.

### Analysis of flowering time genes

We identified nearly 150 genes that had a tendency to increase from the adult to the bolting stages, corresponding to the change from vegetative to reproductive growth. These genes are mainly involved in: transcription regulation pathways, such as for RNA-binding proteins; protein biosynthesis, such as ribosomal protein L1/L13/S7; ubiquitin signaling, such as ubiquitin protein, zinc finger (C3HC4-type RING finger), serine/threonine protein kinase, and glycine-rich protein GRP-3; and flower morphogenesis (MADS-box). In contrast, nearly 220 genes showed a tendency to decrease from the adult to the bolting stages. These genes are mainly involved in glutamine metabolism (such as glycosyltransferase family 14 [GT14] and sugar phosphate permease); the protein phosphatase pathway (such as serine/threonine protein kinase and serine/threonine protein phosphatase); transcription regulation (such as RNA-binding proteins, meprin, and TRAF homology domain-containing protein); protein biosynthesis (such as ribosomal protein L1/L10/L2/L4/L5/S10/L21E/S12/S3AE and zinc finger protein); and some transcription factors (such as MAF1 [MADS AFFECTING FLOWERING 1], CBF2 [C-REPEAT/DRE BINDING FACTOR 2] and TINY [a member of the DREB subfamily A-4 of ERF/AP2 transcription factor family].

*MAF1* was considered a potential flowering inhibitor because it was specifically expressed in the vegetative stages (leaf, rosette, and adult). This result is consistent with Ratcliffe et al. [16] and He et al. [17]. *CBF2* and *TINY* were expressed rarely in the bolting stage and not expressed in the flowering stage.

Seo et al. [18] found that overexpression of cold-inducible *CBF*s could increase expression of FLOWERING LOCUS C (*FLC*), an upstream negative regulator of *SOC1*, thus delaying flowering. In addition, low temperature could induce the expression of the CBFs [19]. Overexpression of the *CBF2* gene also leads to increased freezing tolerance in *Arabidopsis*[20,21]. Because there is crosstalk between cold response and flowering, we hypothesized that decreased expression of *CBF2* was related to the conversion from vegetative to reproductive growth.

We also found some genes that are specifically related to the late flowering of NHCC004. An example is *FLM* (FLOWERING LOCUS M; *CabbageG_a_f_g029765*, homologous with *Bra024350*), a MADS-box transcription factor that is a negative regulator of flowering [22] expressed in the bolting and flowering stages of NHCC004, but not in NHCC001 or NHCC002 (Additional file 2: Figure S8a). The results of qRT-PCR were in accord with this expression trend (Additional file 2: Figure S8b). This suggests that *FLM* may be the reason for late flowering in NHCC004.

Gibberellin-insensitive gene (*CabbageG_a_f_g018551*) was also found in the bolting stage of NHCC004, but not detected in any stages of NHCC001 or NHCC002 (Additional file 2: Figure S8c). The qRT-PCR results for this gene showed lower expression levels in NHCC001 and NHCC002 relative to that of NHCC004 at the bolting stage (Additional file 2: Figure S8d). Many studies have shown that gibberellin is required for flowering in *Arabidopsis* during short days [23,24]. Increased expression of gibberellin-insensitive gene in NHCC004 weakens the role of gibberellin in promoting flowering. Therefore, high expression of flowering suppressor genes may be a reason for late flowering in NHCC004.

**Figure 3:**
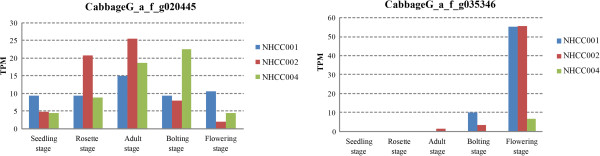
Expression levels of two candidate flowering genes in non-heading Chinese cabbage.

The *FT* (FLOWERING LOCUS T) gene (*CabbageG_a_f_g035346*, homologous with the *Bra004117*, respectively), which promote flowering [25,26], were expressed only at the flowering stage in NHCC004, while they were expressed in the bolting and flowering stages in NHCC001 and NHCC002 (Figure 3). In the circadian pathway of flowering, *FT* was negatively regulated by *ELF3*[27]. Our expression profiling analysis found that the *ELF3* gene (*CabbageG_a_f_g020445*) gradually increased and then decreased in all three accessions. The expression of *ELF3* peaked during the adult stage of NHCC001 and NHCC002 and in the bolting stage of NHCC004. The homologous genes, *ELF4* (*CabbageG_a_f_g013931*) and *ELF6* (*CabbageG_a_f_g052536*), showed the same trend (Additional file 1: Table S2). These results suggest that delayed expression of flowering genes might further explain late flowering in NHCC004.

Specific genes at the flowering stage may be related to the process of flower development and pollination. Our findings indicate that the expression levels of some genes, such as *CabbageG_a_f_g015439* and *CabbageG_a_f_g018658*, were significantly highest at the flowering stage of all three accessions. *CabbageG_a_f_g015439* gene, which encodes for ARK3 protein and is homologous to SLG (S locus glycoprotein), is involved in recognition of self-pollen [28,29]. *CabbageG_a_f_g018658* gene encodes for AGL6 (agamous-like MADS-box protein 6) and functions as a DNA binding and transcription factor. Members of the MADS-box gene family have important roles in flower development, and participate in determining the identity of floral meristems early in flower development and of floral organ primordia later in flower development [30].

### Analysis of cold-tolerance genes

**Figure 4:**
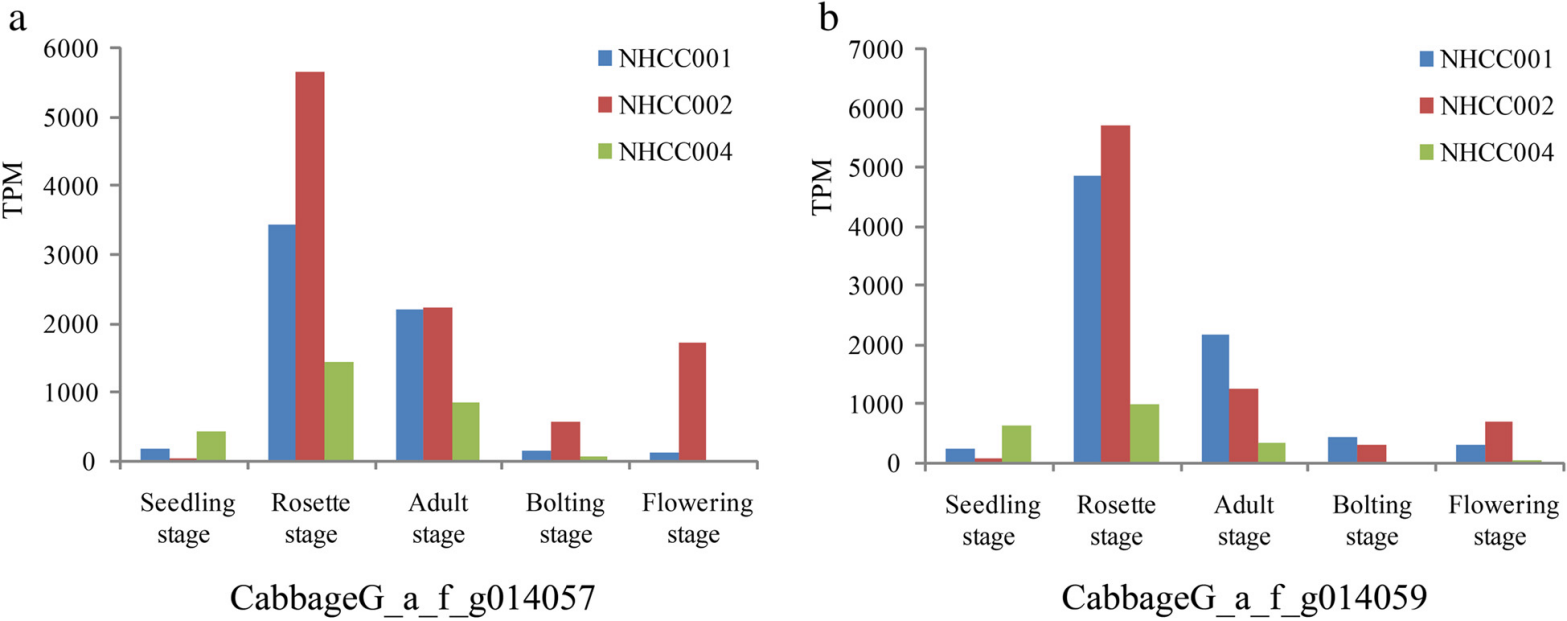
**Cold-tolerance genes identified from differentially expressed genes in non-heading Chinese cabbage. a) ***CabbageG_a_f_g014057; ***b) ***CabbageG_a_f_g014059.*

From expression profiling, we found that two cold-regulated genes (*CabbageG_a_f_g014059* and *CabbageG_a_f_g014057*, homologous with *Bra000265* and *Bra000263*, respectively, of heading Chinese cabbage) showed a higher expression level at the rosette stage (transcripts per million [TPM] > 4000) than at the other four stages (Figure 4).

**Figure 5:**
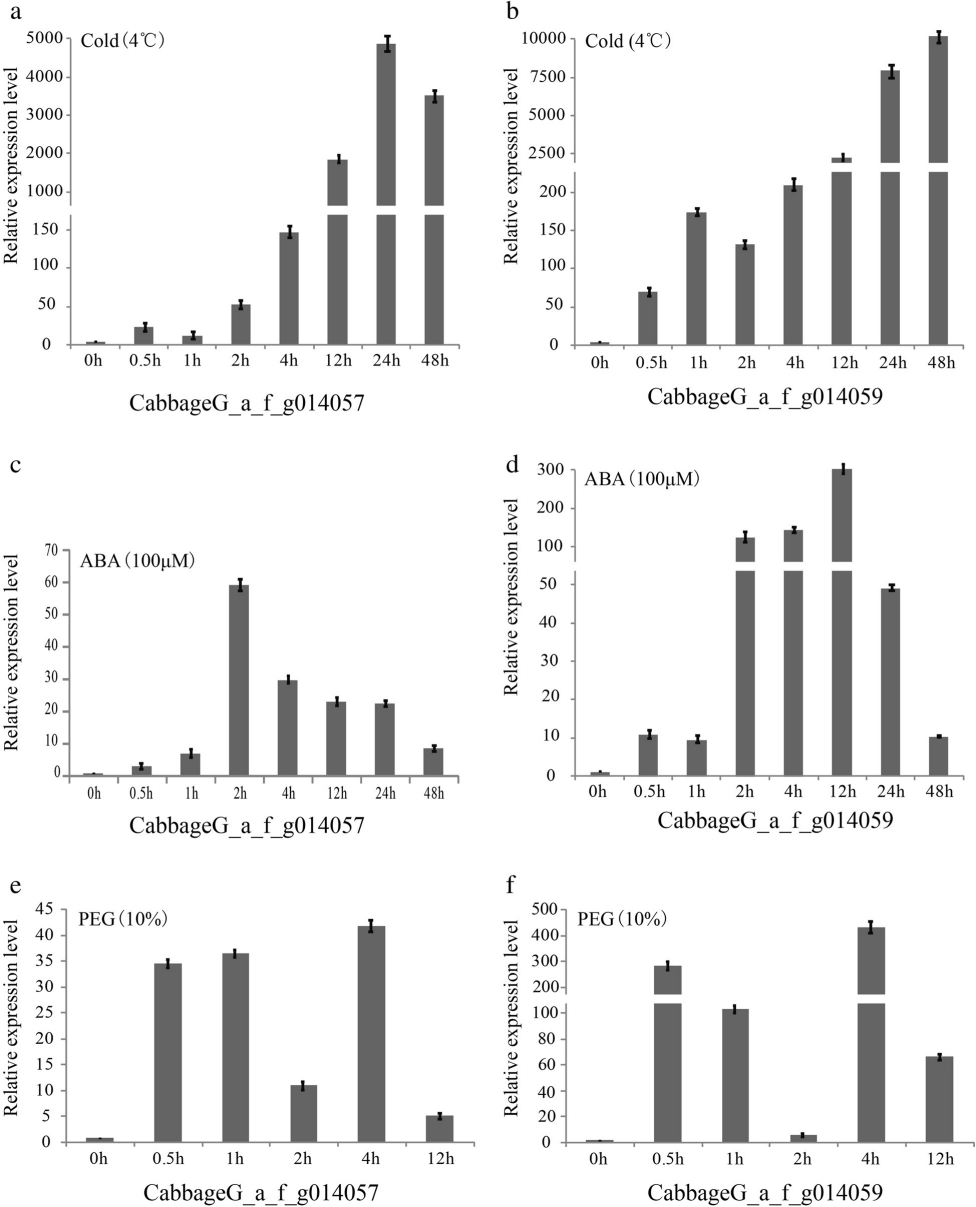
**The relative expression levels of two candidate cold-tolerance genes during treatments. a, b)** Cold treatment; **c, d)** abscisic acid treatment; and **(e, f)** polyethylene glycol. Error bars represent standard errors from three independent replicates.

Weather temperatures fluctuate significantly in Nanjing during autumn and winter. For example, in 2009 the temperature dropped from 10°C to 0°C over five days (from October 15 to October 20). These dramatic changes in the external temperature may be the reason for the high expression of cold-regulating genes. To test this inference, we studied the expression levels of these two genes using quantitative real-time PCR (Figure 5). The results showed that the relative expression values were >1000 after 12 h at 4°C treatment. In addition, the relative expression levels were also changed after abscisic acid (ABA) and polyethylene glycol (PEG) treatments. In general, low temperature, PEG, and ABA crosstalk to activate stress gene expression, and the expression of most cold-related genes is also affected by PEG or ABA treatments. Therefore, we suggest that the high expression of these genes was closely linked to cold resistance in non-heading Chinese cabbage.

### Analysis of self-incompatibility genes

Three genes, *CabbageG_a_f_g006792*, *CabbageG_a_f_g011856*, and *CabbageG_a_f_g039867*, were expressed at all five stages and specifically existed only in NHCC002 (Additional file 2: Figure S9). These genes encode serine/threonine protein kinase, NAC domain-containing protein 82, and dynein light chain type 1 family protein, respectively. Among them, *CabbageG_a_f_g006792* and *CabbageG_a_f_g011856* showed significantly decreased levels at the bolting and flowering stages compared with vegetative stages. However, the *CabbageG_a_f_g039867* transcript increased at the bolting stage and declined at the flowering stage. In the process of self-pollination, serine/threonine protein kinases, such as S-locus receptor kinase (SRK) and M-locus protein kinase (MLPK), are involved in recognition and autophosphorylation in self-incompatible signaling pathways [31-33]. Thus, changes in the expression levels of *CabbageG_a_f_g006792* could influence the activation of serine/threonine protein kinase, thereby affecting the recognition and rejection of self-pollen. NAC domain-containing proteins are plant-specific transcriptional factors involved in regulating several plant developmental processes, such as flower and embryo development [34-36]. Thus, these specifically expressed genes might be related to self-incompatibility of NHCC002.

We also found that *CabbageG_a_f_g031080* had higher transcripts at all stages in accessions NHCC001 and NHCC004, while it was expressed at low levels at the bolting and flowering stages in NHCC002 (Additional file 2: Figure S9). This gene encodes ribosomal protein L13, which is involved in the assembly of proteins. The downregulation of ribosomal protein L13 might be related to the suppression of self-pollen development in NHCC002. Compared with NHCC001 and NHCC004, 46 genes of the NHCC002 accession showed lower transcript levels, with zero TPM, even during flowering.

Interestingly, some genes encoded vesicle coat complex and ATEXO70H7, which are related to secretory protein trafficking and polarized exocytosis [37,38]. Exo70A1 participates in the growth of pollen tube tips and has been identified as a negative regulator in the *Brassica* self-incompatibility response [39,40]. We found that the expression levels of vesicle coat complex and ATEXO70H7 were higher in NHCC001 and NHCC004 compared with NHCC002 accession, given that the expression levels of these proteins were nil at the flowering stage. The low abundance of vesicle coat complex and ATEXO70H7 implied that they might have similar functions as negative regulators in the self-incompatibility response of NHCC002.

### Analysis of leaf color genes

We analyzed the genes associated with leaf color. These genes have an important role in the control of chlorophyll biosynthesis, chloroplast structure, and plant development. Moreover, they might affect crop yields by regulating photosynthesis. Therefore, it is crucial for improving crop production to identify leaf-color related genes and uncover the genetic basis of the leaf color trait. The chlorophyll content of the leaves was measured using a portable chlorophyll meter (SPAD-502Plus, Konica Minolta). The measurement results showed that the chlorophyll indices of NHCC001 and NHCC002 were significantly different at the rosette stage. After analyzing the genes involved in the chlorophyll gene (KO00860) pathway of these two accessions, we found that the light leaf color of NHCC002 is most likely due to decreased chlorophyll synthesis, perhaps resulting from lower chlorophyllase activity [41,42].

The *CabbageG_a_f_g026085* gene, which encodes the chloroplastic protein FLUORESCENT IN BLUE LIGHT, was also expressed at lower levels in NHCC002. Furthermore, qRT-PCR showed the same expression pattern as the expression profile (Additional file 2: Figure S10). It is involved in the regulation of chlorophyll biosynthesis and might be a negative regulator of tetrapyrrole biosynthesis in chloroplasts [43,44]. This result implied that *CabbageG_a_f_g026085* could also be a candidate gene related to chlorophyll biosynthesis in non-heading Chinese cabbage.

### DEGs in the genomic colinear blocks between *B. rapa* and *A. thaliana*

**Figure 6:**
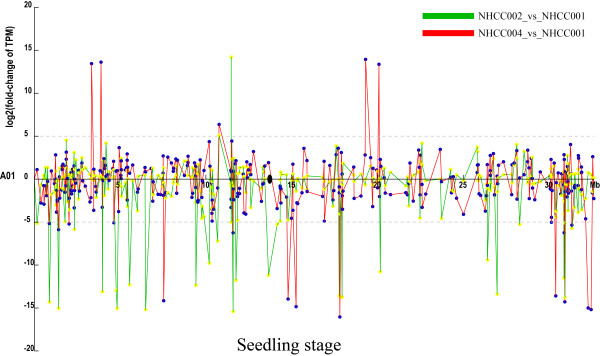
TPM fold-change of differentially expressed genes among three accessions on chromosome 1 in the seedling stage.

In our expression profile, 29 101 genes were expressed and 28 638 (98.4%) of the expressed genes were mapped to 10 chromosomes. In these expressed genes, 15 830 (54.4%) were identified as DEGs of different accessions or developmental stages, and 15 567 (98.3%) genes were located on the 10 chromosomes. To show the up- and downregulation of DEGs in a more intuitive way, we labeled the DEGs on each chromosome with the regulation information (Figure 6, Additional file 2: Figure S11).

**Figure 7:**
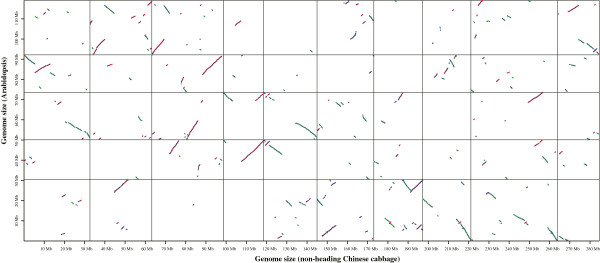
**Whole-genome colinear blocks between non-heading Chinese cabbage and *Arabidopsis thaliana*.** Syntenic blocks are formed by red or green dots representing the best hits across any two chromosomes in the same or opposite direction, respectively. The blue dots represent the differentially expressed genes present in the colinear blocks.

A total of 581 colinear blocks were identified between the genomes of non-heading Chinese cabbage and *Arabidopsis*. Finally, 369 (63.5%) colinear blocks were obtained after removing the blocks that contained fewer than 10 genes from consideration. Of the 15 830 DEGs, nearly half of them (7504, 47.4%) were located in the colinear blocks (Figure 7). In non-heading Chinese cabbage and heading Chinese cabbage, 710 colinear blocks were identified. Four hundred and twelve (58.0%) colinear blocks were obtained after removing the blocks containing fewer than 10 genes. A total of 23.1% (3652) of the DEGs were identified in the colinear blocks (Additional file 2: Figure S12).

To further characterize the relationships among non-heading Chinese cabbage, heading Chinese cabbage, and *Arabidopsis* we analyzed the paralogous and orthologous genes among them. There were 31 322, 20 770, and 23 171 paralogous gene pairs in the entire genomes of non-heading Chinese cabbage, heading Chinese cabbage, and *Arabidopsis*, respectively. For the orthologous genes, there were 46 716 gene pairs between non-heading Chinese cabbage and *Arabidopsis*, whereas there were 64 975 gene pairs between non-heading Chinese cabbage and heading Chinese cabbage. Furthermore, we investigated the number of differentially expressed paralogous genes that existed in non-heading Chinese cabbage, and we found that 4092 (25.8%) genes had a paralogous gene in non-heading Chinese cabbage (Additional file 1: Table S6). Of these genes, 1,960 had only one paralog. The number of the paralogs was >10 for 117 DEGs, and >50 for 16 DEGs.

Most of the genes that had >50 paralogs belonged to the non-long terminal repeat retroelement reverse transcriptase. We used the Pfam program (http://pfam.sanger.ac.uk/) [45] to identify 3840 paralogs that belonged to 9183 DEGs. Among these paralogs, most encoded proteins were leucine-rich repeat protein, protein kinase domain protein, WD domain, and G-beta repeat (Additional file 2: Figure S13).

We also analyzed orthologous pairs of DEGs, identifying 13 932 genes that were orthologous between the non-heading Chinese cabbage and heading Chinese cabbage. Of these, 9012 genes had one ortholog in heading Chinese cabbage, 314 genes had >10 orthologs in heading Chinese cabbage, and one gene (*CabbageG_a_f_g017292*) had 54 orthologs in heading Chinese cabbage. While there was a total of 11 512 orthologs (72.7%) between non-heading Chinese cabbage and *Arabidopsis* (Additional file 1: Table S7), 7368 genes had only one ortholog in *Arabidopsis*, which decreased to 199 when the number of orthologs was >10. Interestingly, the same gene (*CabbageG_a_f_g017292*) also had a relatively high number of orthologs (i.e., 56) in *Arabidopsis*. The same gene also had 37 paralogs in non-heading Chinese cabbage. Although the explanation for the high number of copy number variations for this gene is unknown, we inferred that it might affect plant growth. Therefore, we annotated its function, which revealed that it was a disease resistance protein (TIR-NBS-LRR class).

## Conclusions

In our analysis, we identified numerous DEGs related to important agricultural traits. By comparing cultivars and developmental stages, we found many genes associated with flowering time, self-incompatibility, cold-tolerance, and leaf color. Although the functions of most of the other DEGs are not known, this study will further our understanding of the expression pattern of these genes and genetic improvement of non-heading Chinese cabbage or other cruciferous vegetables, as well as basic biological research. In particular, clarification of the regulatory networks involved in flowering will contribute to the cultivation of new late-flowering varieties, which can provide a wealth of resources for breeding. This detailed analysis of the expression profiles of non-heading Chinese cabbage provides the first comprehensive review of the expression patterns of five development stages, and has unveiled numerous candidate genes that may underlie morphological and genetic polymorphisms of non-heading Chinese cabbage.

## Materials and methods

### Sample preparation

The non-heading Chinese cabbage accessions NHCC001, NHCC002, and NHCC004 used for expression profiles were cultivated in the field under a non-controlled environment. RNA was extracted at the five important plant developmental stages: seedling, rosette, adult, bolting, and flowering. During the first three stages, RNA for expression profiles was extracted from leaves. The RNA of the bolting stage was extracted from an equal mixture of leaf and buds, while leaves and flowers were used for RNA extractions during flowering. All the RNA was extracted in accordance with the manufacturer’s instructions for the RNeasy plant mini kit (Qiagen).

### Digital gene expression tag profiling

The expression levels of genes for the three accessions at the five development stages were obtained using digital gene expression-tag profiling methods, as in a previous report [15]. The number of times that a unique tag sequence was detected represents the quantitative expression of the corresponding transcript in tissues.

The Bowtie program (http://bowtie-bio.sourceforge.net/index.shtml) was used to map sequencing reads to the non-heading Chinese cabbage genome [46]. Finally, high quality clean tags were compared with the genome sequences of non-heading Chinese cabbage and the expression level of each gene was quantified as TPM [47].

To evaluate the mRNA expression characteristics, we analyzed the TPM of each identified gene. The highly expressed genes (defined as TPM > 100) accounted for >50% of the total expression tags from all genes (Additional file 2: Figure S14a) in the NHCC001 accession. The number of genes with a TPM < 10 was ~55% of all genes, while the number of genes with a TPM > 80 only accounted for ~10% of all genes (Additional file 2: Figure S14b). Statistical analysis of the other two accessions showed similar patterns, illustrating that most genes were expressed at a low level, and only a handful of genes expressed at high levels accounted for the majority of tag reads. This is in accord with the heterogeneous and redundant features of mRNA expression.

We also performed saturation analyses of sequencing data. The data show that the more sequencing tags that a gene had, the more likely it was to be expressed in a certain range. When the number of tags reached a threshold, the number of expressed genes approached saturation. Our analysis shows that the number of expressed genes was nearly saturated when tag number was >3 million (Additional file 2: Figure S14c). The lowest number of tags obtained in our study was 3.40 million for the rosette stage of NHCC004. Therefore, our sequencing reads had reached saturation for all the sample stages, assuring that most of the expressed genes during plant growth and development were detected in our study.

### Identification of DEGs

The DEGs were identified mainly as previously described [48]. The expressed genes were identified using IDEG6 software [49], and a general chi-squared test was used to test the hypothesis. The false discovery rate was used to correct the *P*-value [50], and the fold changes were also calculated for identification of the differentially expressed genes. To avoid the potential noise signal from high-throughput sequencing, absolute fold change ≥2.0 and a false discovery rate <0.01 were used to define the DEGs, including the upregulated and downregulated genes. The differential expression levels of the genes at the five stages of the three accessions were compared and visualized through scatter plots drawn by Perl scripts, and their expression pattern was displayed using the heatmap function in the Cluster program (http://bonsai.hgc.jp/~mdehoon/software/cluster/software.htm) and visualized using Tree View software (http://jtreeview.sourceforge.net/) [51]. An interaction network of the DEGs was constructed using Cytoscape software (http://www.cytoscape.org/) according to the expression level of the genes [52]. The numbers of specific and common DEGs were plotted using the Venn diagram function in the R software package [53].

### Plant materials, growth conditions, and stress treatments

To verify the two candidate cold-related genes, the non-heading Chinese cabbage cultivar ‘Suzhouqing’ (NHCC001) was used for quantitative real-time PCR. Seeds were grown in pots containing a soil: vermiculite (3:1) mixture in a controlled-environment growth chamber programmed for 16/8 h at 25/20°C for day/night, relative humidity of 55-60%. At the rosette stage, they were transferred to growth chambers set at 4°C under the same light intensity and day length as the cold treatments. The leaf samples were collected at 0, 0.5, 1, 2, 4, 12, 24, and 48 h after cold treatment. At the same time for acclimation, some plants were cultured in 1/2 Hoagland’s solution in plastic containers, with pH at 6.5. After 5 days of acclimatization, plants were cultured in 100 μМ abscisic acid, 10% polyethylene glycol, or left untreated. Leaf samples were collected 0, 0.5, 1, 2, 4, 12, 24, and 48 h after these treatments and then frozen in liquid nitrogen and stored at −70°C until further analysis.

### RNA isolation and quantitative real-time PCR analysis

Total RNA was isolated from leaves using an RNA kit (Tiangen, China) in accordance with the manufacturer’s instructions. The RNA was reverse transcribed into cDNA using the Prime Script RT reagent kit (TaKaRa, Japan). The actin gene (*AF111812*) was used as an internal control to normalize the expression level of the target gene among different samples [54]. The specific primers were designed according to the target gene sequences by Primer 5.0 software (Additional file 1: Table S8). Quantitative real-time PCR assays were performed with three biological and three technical replicates. Each reaction was performed in a 20-μL reaction mixture containing diluted cDNA as template, SYBR Premix Ex Taq (2×) (TaKaRa, Japan), and gene-specific primers. Quantitative real-time PCR was performed using MyiQ Single-Color Real-Time PCR Detection System (Bio-rad, Hercules, CA) with the following cycling profile: 94°C for 30 s, and then 40 cycles at 94°C for 10 s, 58°C for 30 s, and then a melting curve (61 cycles at 65°C for 10 s) was generated to check the specific amplification. The relative quantitative method was employed to analyze the relative gene expression level. RNA levels were expressed relative to that of the actin gene (*AF111812*) as 2^–ΔΔCT^, where Ct is the cycle threshold, in accordance with previous studies [55].

### Functional annotation and pathway analysis

The annotations of DEGs in non-heading Chinese cabbage were obtained by searching the protein databases Iprscan (http://www.ebi.ac.uk/Tools/pfa/iprscan/), UniProtKB (http://www.ebi.ac.uk/uniprot/) [56], TrEMBL (http://www.ebi.ac.uk/uniprot/TrEMBLstats/) [57], GO (http://www.geneontology.org/) [58], and KEGG (http://www.genome.jp/kegg/) [59] and the annotations obtained from these five protein databases were integrated using Perl script. In addition, the biological processes and functions of DEGs were analyzed using the COG (http://www.ncbi.nlm.nih.gov/COG/) [60], and GO databases. The COG database represents major phylogenetic lineages, and each COG consists of individual proteins or groups of paralogs from at least 3 lineages.

### Mapping differentially expressed genes on the draft genome

The distribution of all predicted genes, expressed genes, and differentially expressed genes on chromosomes were visualized using Perl scripts, and differently colored lines represented each gene dataset. The orthologous and paralogous genes were identified using OrthoMCL software (http://www.orthomcl.org/cgi-bin/OrthoMclWeb.cgi) [61], and the copy number of these genes was calculated using Perl scripts. The syntenic relationships between species was constructed by McScan (MATCH_SCORE: 40, MATCH_SIZE: 5, GAP_SCORE:-2, EXTENSION_DIST: 40, E_VALUE: 1e-05; http://chibba.agtec.uga.edu/duplication/mcscan/) [62]. The all-against-all BLASTP comparison provided the E-value and the pairwise gene information for protein clustering. Paired segments were extended by identifying clusters of genes. This method was used to build the genome synteny blocks of non-heading Chinese cabbage compared with heading Chinese cabbage and *Arabidopsis*. Furthermore, we filtered the synteny blocks that had <10 genes to obtain improved collinear analysis. Finally, the DEGs that were located in the synteny blocks were marked according to the physical position, and the collinear blocks plus the marked DEGs were plotted using Perl scripts.

## Competing interests

The authors declare that they have no competing interests.

## Authors’ contributions

The study was conceived by XS and XH. XH and XS collected the dataset of non-heading Chinese cabbage. XS contributed to data analysis, bioinformatics analysis, and manuscript preparation. XS and YL participated in writing the manuscript. All authors contributed to revising the manuscript. All authors read and approved the final manuscript.

## Supplementary Material

Additional file 1: Table S1 Expression profile data at five development stages of three non-heading Chinese cabbage accessions. **Table S2.** Expression values at five development stages of three non-heading Chinese cabbage (TPM). **Table S3.** The number of differentially expressed genes among different evelopmental stages for each accession. **Table S4.** Differentially expressed genes among three accessions at the same developmental stage. **Table S5.** Statistical analysis of the differentially expressed genes between NHCC001 and NHCC002 at the seedling stage. **Table S6.** Number of paralogous genes in differentially expressed genes of non-heading Chinese cabbage. **Table S7.** Number of orthologous genes in differentially expressed genes of non-heading Chinese cabbage compared with *Arabidopsis* and heading Chinese cabbage. **Table S8.** Primer sequences used for quantitative real-time PCR amplification of actin and two cold-tolerance genes. Click here for file

Additional file 2: Figure S1 Number of expressed genes in each developmental stage. **a)** NHCC001; **b)** NHCC002; **c)** NHCC004. **Figure S2.** Numbers of expressed genes for the three accessions at each developmental stage. **a)** Seedling; **b)** rosette; **c)** adult; **d)** bolting; **e)** flowering. **Figure S3.** Numbers of differentially expressed genes for the five developmental stages in accessions NHCC002 and NHCC004. **Figure S4.** Genes differentially upregulated or downregulated in the seedling stage among the accessions. The red dots represent the upregulated genes, and the green dots represent the downregulated genes. **Figure S5.** Cluster graph of self-incompatibility candidate genes, in TPM. **Figure S6.** GO annotation of the differentially expressed genes. **Figure S7.** COG of the differentially expressed genes. **Figure S8.** Expression levels of two candidate flowering genes. **a, c)** Transcription per million, by expression profile; and **(b, d)** relative expression levels, by qRT-PCR. **Figure S9.** Self-incompatibility genes identified from differentially expressed genes in non-heading Chinese cabbage. **Figure S10.** The expression levels and pathways of chlorophyll genes involved in non-heading Chinese cabbage. **Figure S11.** TPM fold-changes of differentially expressed genes among the three accessions at each stage on chromosome. (I.e., on chromosome 1 for the rosette, adult, bolting, and flowering stages.). **Figure S12.** Whole-genome colinear blocks between non-heading Chinese cabbage and heading Chinese cabbage. Syntenic blocks are formed by red or green dots representing the best hits across any two chromosomes in the same or opposite directions, respectively. The blue dots represent the differentially expressed genes present in the colinear blocks. **Figure S13.** Pfam domain annotation for the differentially expressed genes belonging to the paralogous genes and other differentially expressed genes. **Figure S14.** Statistical analysis of expression profile data. **a)** Distribution charts of TPM; **b)** statistical charts for the gene numbers in each TPM interval region; **c)** sequencing saturation analysis chart. Click here for file
